# Supplementary material for: Benchmarking splice variant prediction algorithms using massively parallel splicing assays
Source: Genome Biol. 2023 Dec 21;24:294. doi: 10.1186/s13059-023-03144-z (PMC10734170; doi:10.1186/s13059-023-03144-z)
Supplement: Supplementary file 1 — Additional file 1: Supplementary information. This additional file contains the supplementary figures and descriptions accompanying this manuscript. [file 13059_2023_3144_MOESM1_ESM.pdf]

Supplementary Material

Benchmarking splice variant prediction algorithms using massively parallel splicing assays

Cathy Smith[1,2], Jacob O. Kitzman[1,2*]

Affiliations
[1]Department of Computational Medicine and Bioinformatics, University of Michigan Medical School, Ann Arbor, MI 48109, USA
[2]Department of Human Genetics, University of Michigan Medical School, Ann Arbor, MI 48109, USA
*Correspondence to kitzmanj@umich.edu

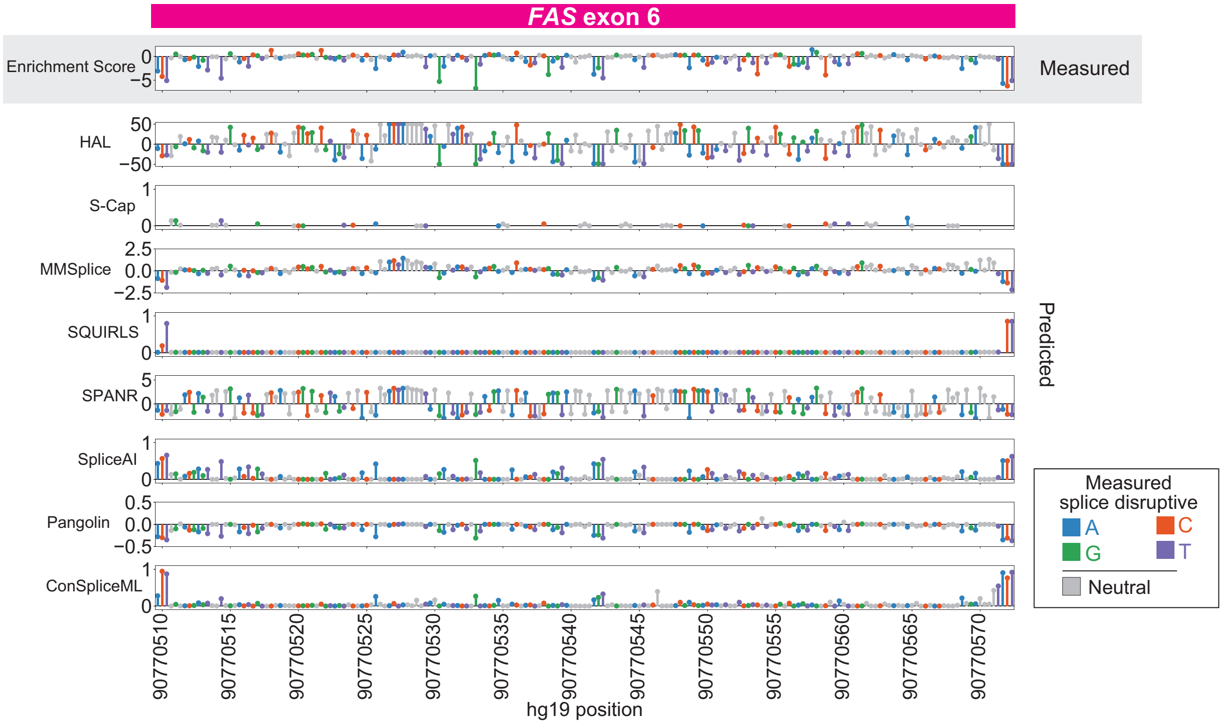

Supplementary Figure 1. Splicing effect map and bioinformatic predictions for *FAS* exon 6. MPSA measured enrichment score of *FAS* exon 6 (gray, top panel; increased skipping – negative values, increased inclusion – positive values), along with bioinformatic predictions (subsequent panels), with splice effects/predictions plotted by variant position. Each lollipop denotes one variant, shaded by effect in MPSA (gray: neutral, colors: SDVs, shaded by mutant base).

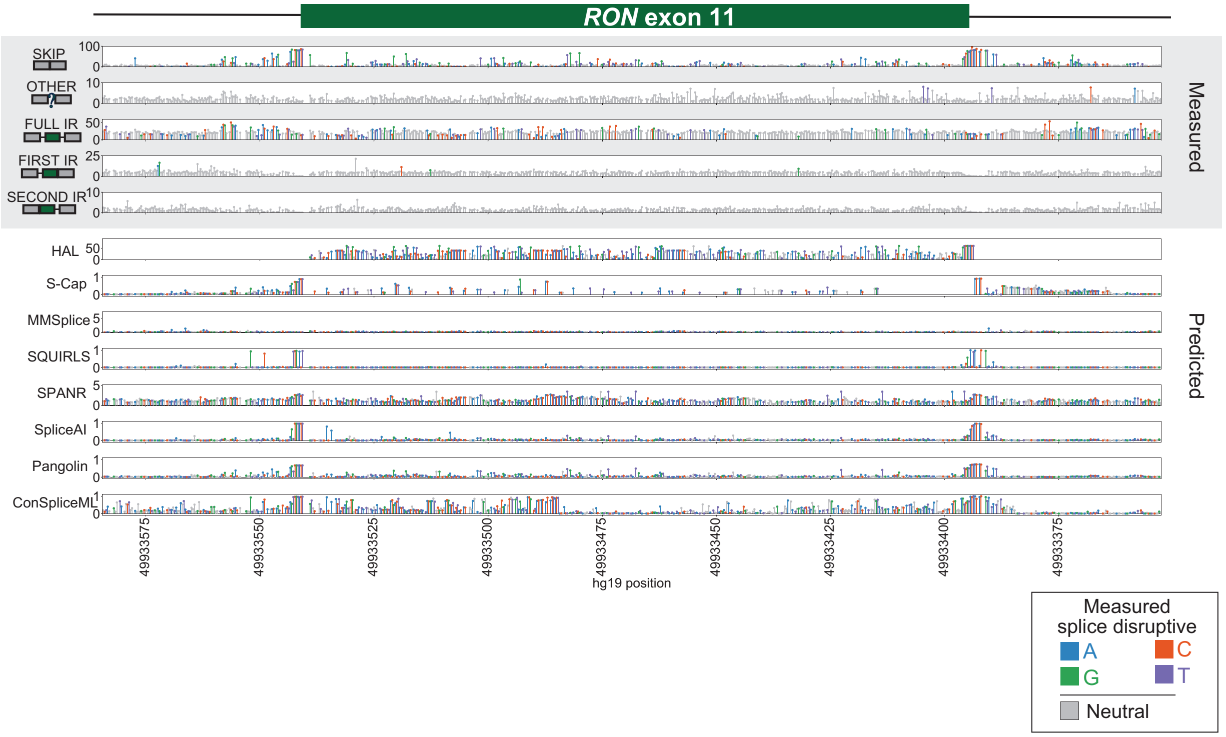

Supplementary Figure 2. Splicing effect map and bioinformatic predictions for RON exon 11. MPSA measured percent usage for different splicing outcomes at RON exon 11 (gray, top panel): skipping, other isoforms, full intron retention (“FULL IR”), first intron retention (“FIRST IR”), and second intron retention (“SECOND IR”), along with bioinformatic predictions (subsequent panels), with splice effects/predictions plotted by variant position. Each lollipop denotes one variant, shaded by effect in MPSA (gray: neutral, colors: SDVs, shaded by mutant base).

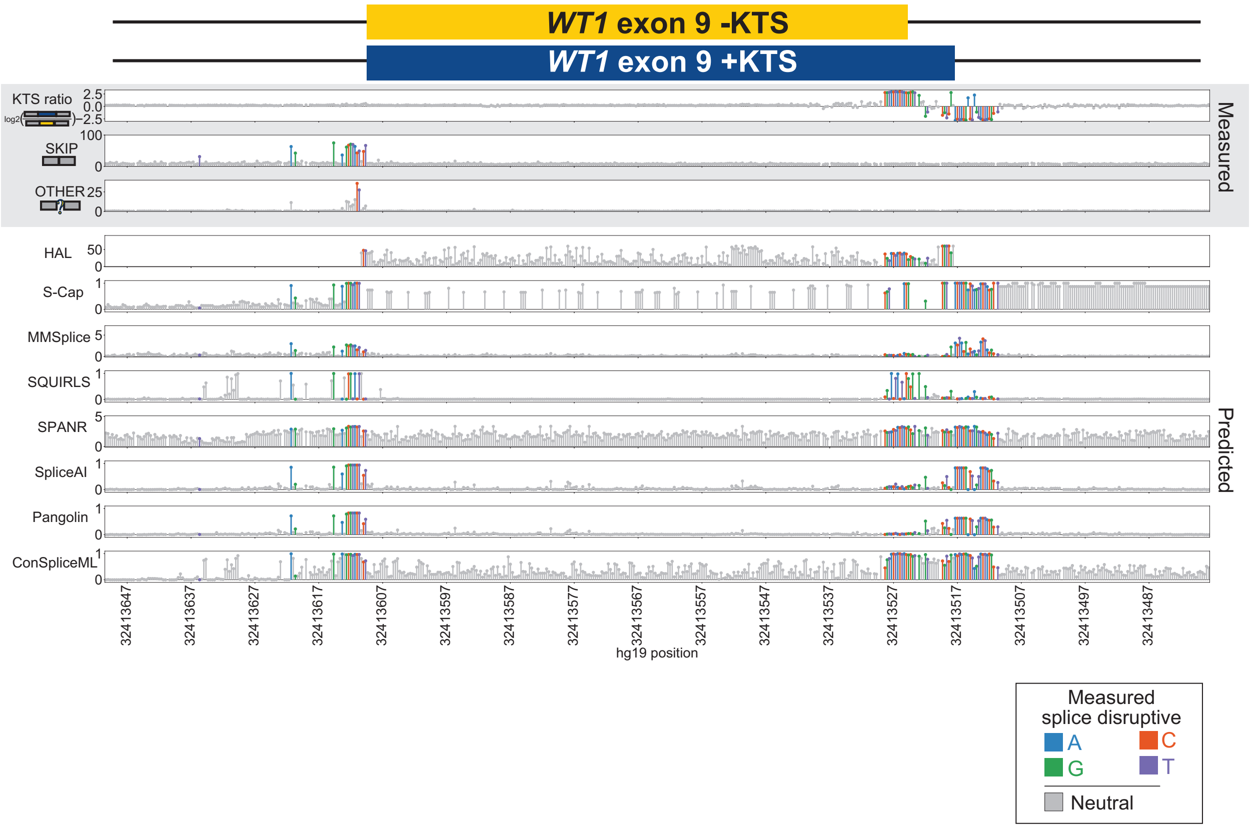

Supplementary Figure 3. Splicing effect map and bioinformatic predictions for *WT1* exon 9. MPSA measurements for different splicing outcomes at *WT1* exon 9 (gray, top panel): log2ratio(%KTS+/%KTS-), percent exon skipping, and percent other isoforms, along with bioinformatic predictions (subsequent panels), with splice effects/predictions plotted by variant position. Each lollipop denotes one variant, shaded by effect in MPSA (gray: neutral, colors: SDVs, shaded by mutant base).

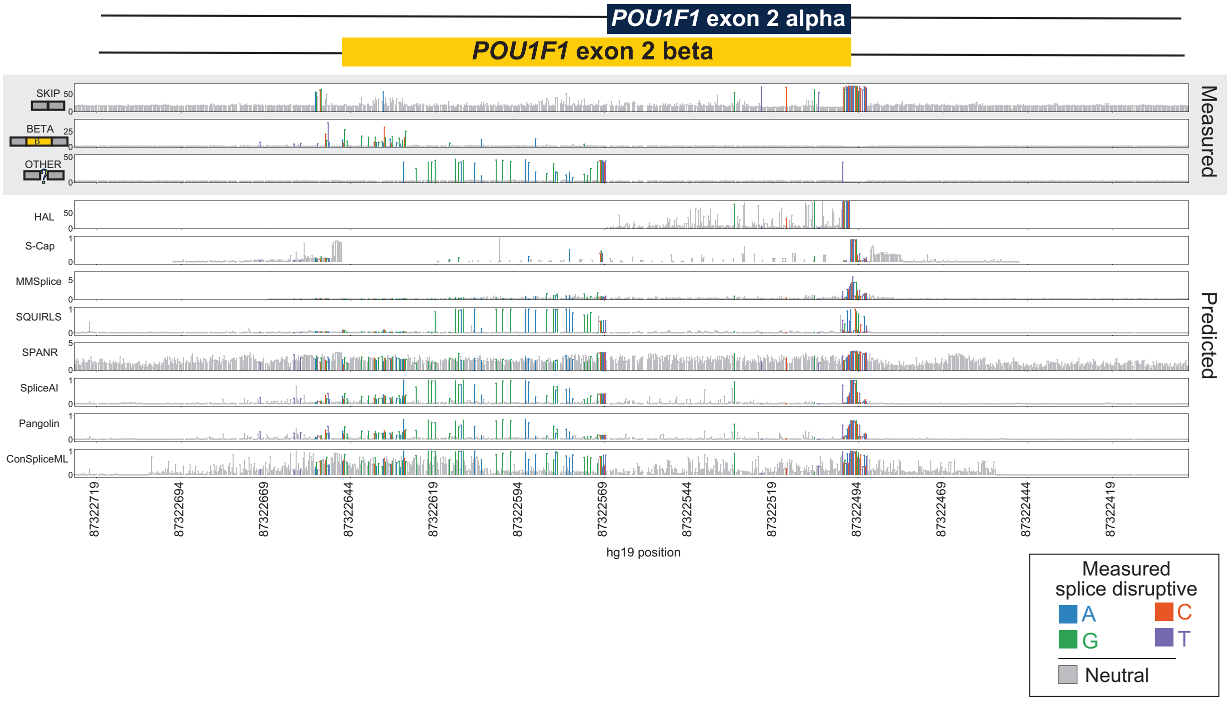

Supplementary Figure 4. Splicing effect map and bioinformatic predictions for POU1F1 exon 2. MPSA measured percent usage for different splicing outcomes at POU1F1 exon 2 (gray, top panel): exon skipping, exon 2 beta, and other isoforms, along with bioinformatic predictions (subsequent panels), with splice effects/predictions plotted by variant position. Each lollipop denotes one variant, shaded by effect in MPSA (gray: neutral, colors: SDVs, shaded by mutant base).

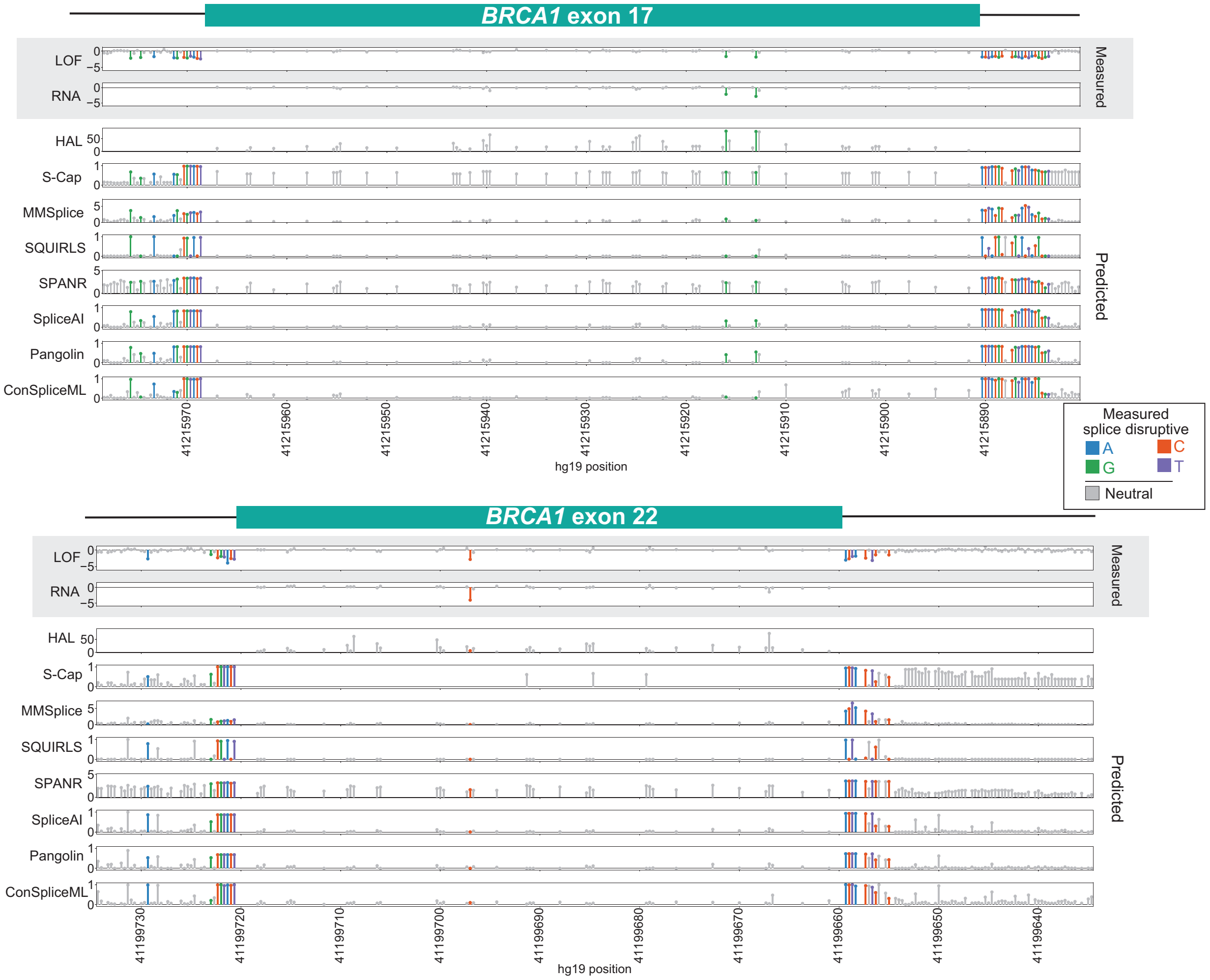

Supplementary Figure 5. Splicing effect map and bioinformatic predictions for *BRCA1* exons. SGE measurements for two representative *BRCA1* exons (exons 17 and 22; in gray, top panel): log2-ratio function score and log2ratio RNA score, along with bioinformatic predictions (subsequent panels), with splice effects/predictions plotted by variant position. Each lollipop denotes one variant, shaded by effect in SGE (gray: neutral, colors: SDVs, shaded by mutant base). Missense and stop gained variants are excluded.

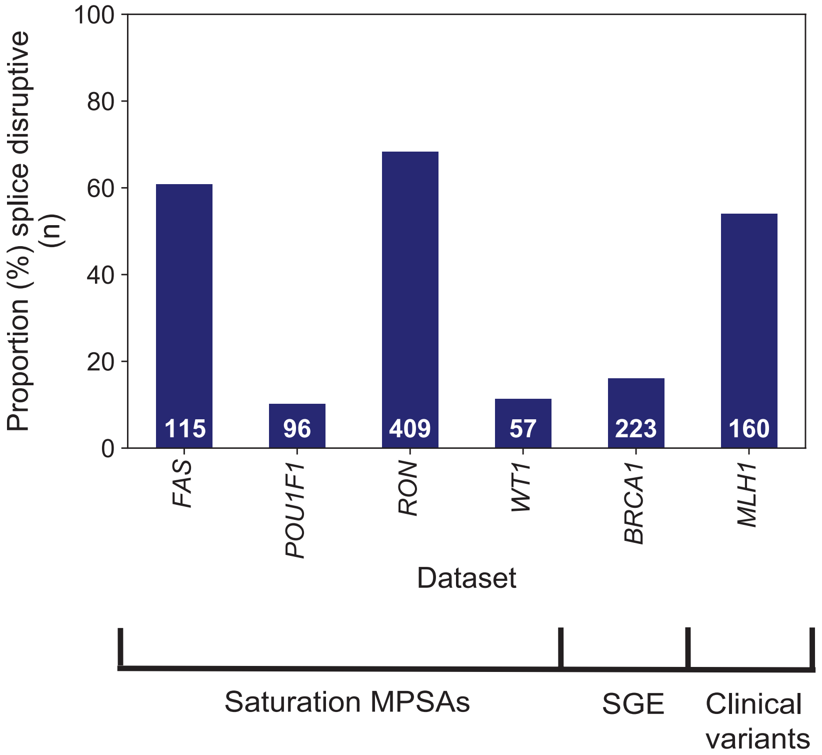

Supplementary Figure 6. Proportion of splice disruptive variants (SDVs) within benchmarked datasets. Bar plot showing the proportion of SDVs out of all measured variants (y-axis) within the saturation MPSAs (*FAS*, *POU1F1*, *RON*, *WT1*), SGE (*BRCA1*), and clinically curated variant set (*MLH1*) (x-axis). Numbers on each bar display the count of splice disruptive SNVs per dataset.

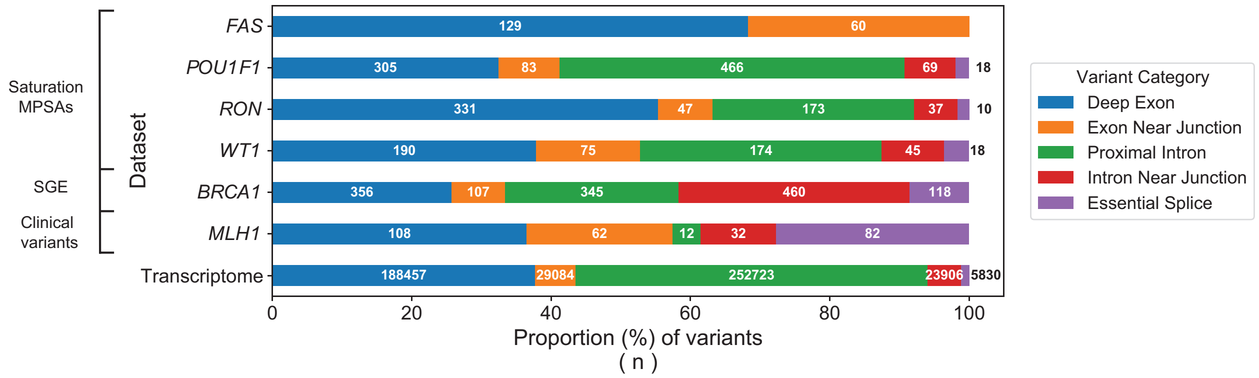

Supplementary Figure 7. Breakdown of benchmark and background variant sets by variant class. Proportions (x-axis) of variant category (color) deep exon (blue) in each benchmark variant dataset. Datasets are grouped by study type (MPSAs, SGE, and clinical variants), and ‘transcriptome’ denotes the random background set of variants. Variant categories are defined and shaded as in **Figure 1B**. Numbers of each bar indicate count of each type of variant per dataset.

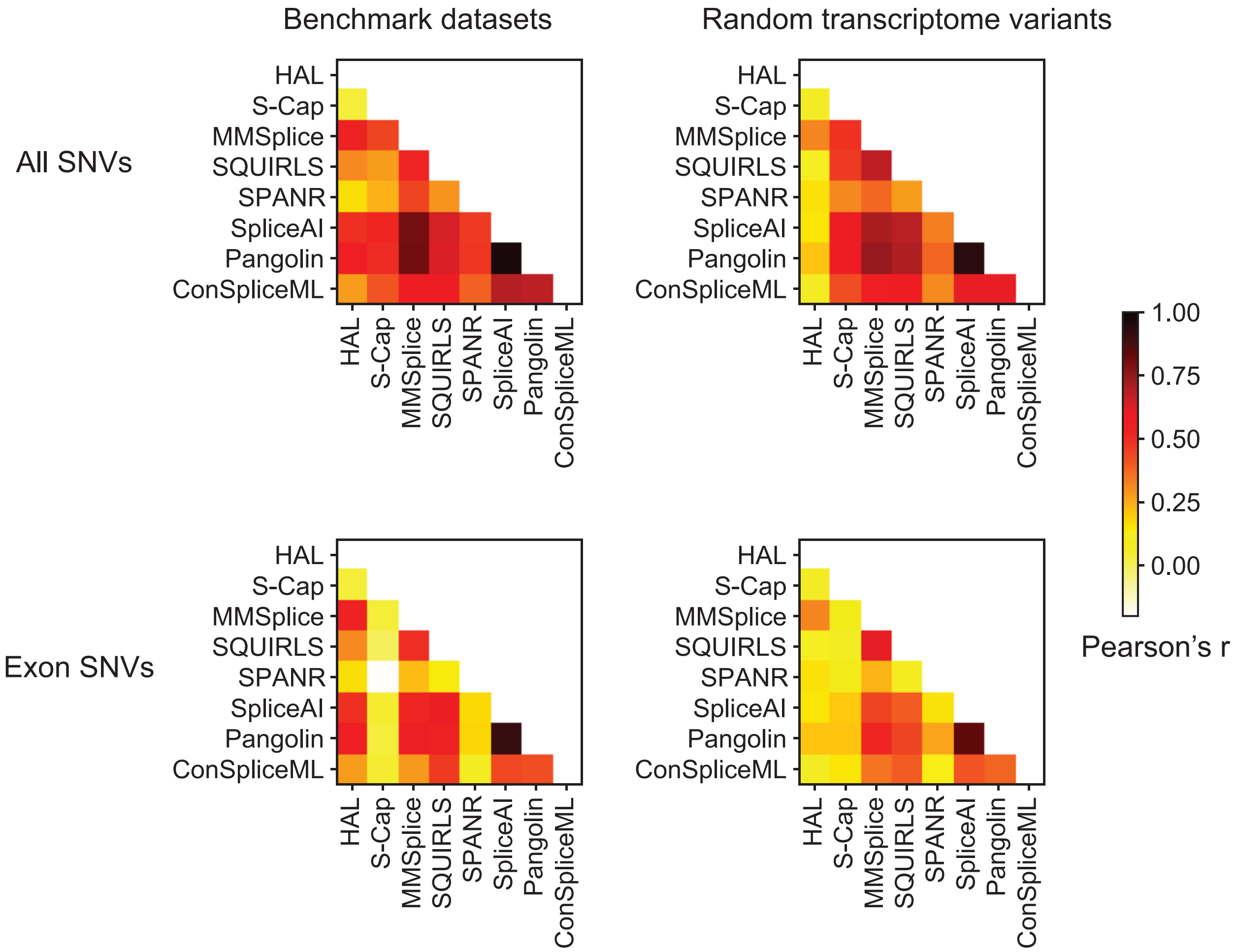

Supplementary Figure 8. Correlations among bioinformatic algorithms. Heatmaps of Pearson correlations between scores from eight bioinformatic algorithms across benchmarked variants (left column) and randomly selected 'background set' variants (right column). Top row shows correlations across all variants; bottom row shows correlations over only exonic variants.

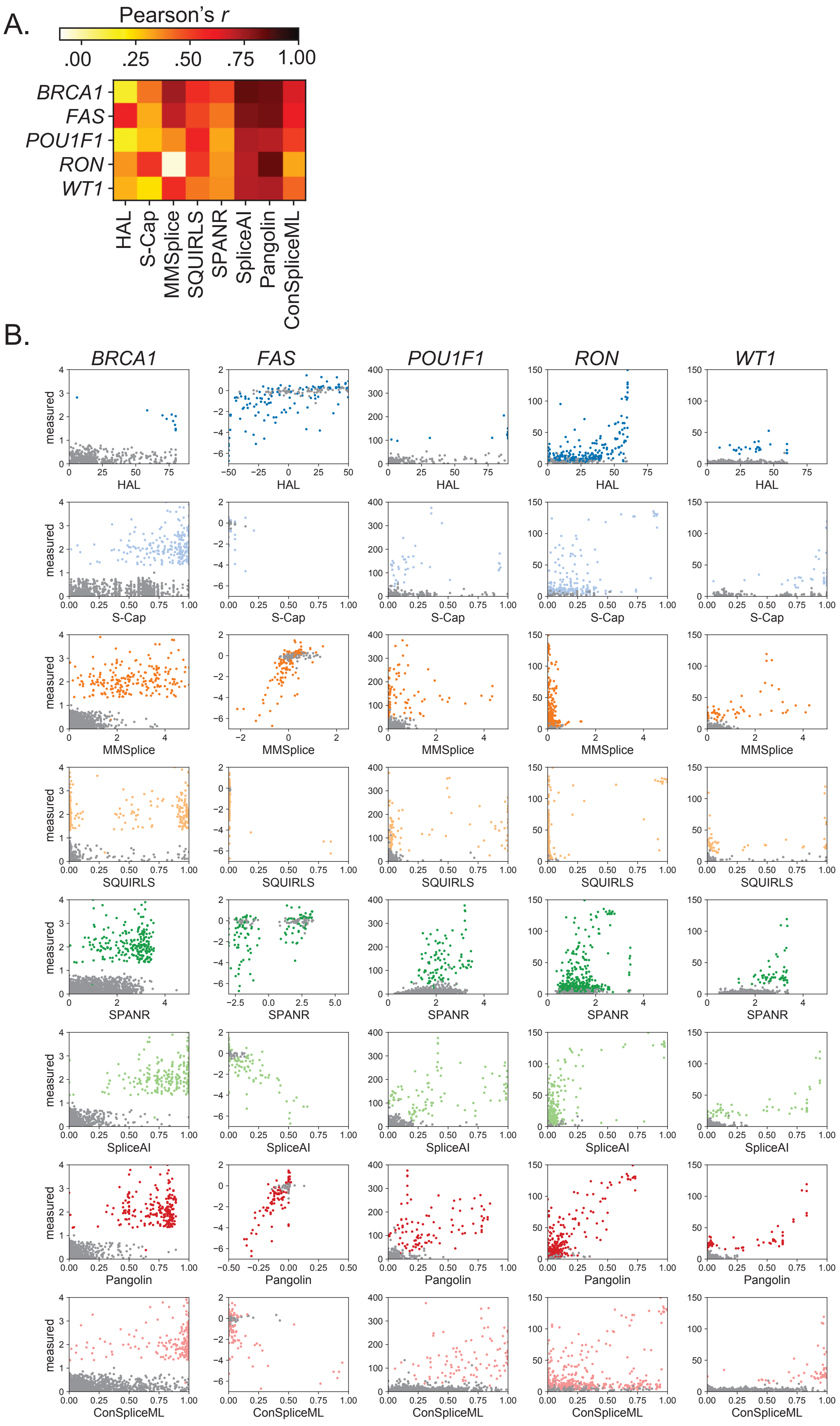
A.
Pearson's r
.00
.25
.50
.75
1.00
BRCA1
FAS
POU1F1
RON
WT1
HAL
S-Cap
MMSplice
SQUIRLS
SPANR
SpliceAI
Pangolin
ConSpliceML
B.
BRCA1
FAS
POU1F1
RON
WT1
measured
HAL
S-Cap
MMSplice
SQUIRLS
SPANR
SpliceAI
Pangolin
ConSpliceML

Supplementary Figure 9. Correlations between bioinformatic algorithms' scores and MPSA measurements. (A) Heatmap showing the Pearson's correlations between bioinformatic algorithms (x-axis) and MPSA-measured effects (y-axis). MPSA measurements were processed as described in Methods (per-variant, the maximum absolute value of *z* score across isoforms was taken for *POU1F1, RON,* and *WT*), and for algorithms reporting a score, the absolute value of the score was taken. *MLH1* SNVs are omitted as they were curated across many different studies and do not have measurements beyond classification as deleterious/neutral. **(B)** Scatterplot of all variants showing measured effect (y-axis) and predicted effect (x-axis); gray points denote splice neutral variants and shaded points are splice-disruptive variants.

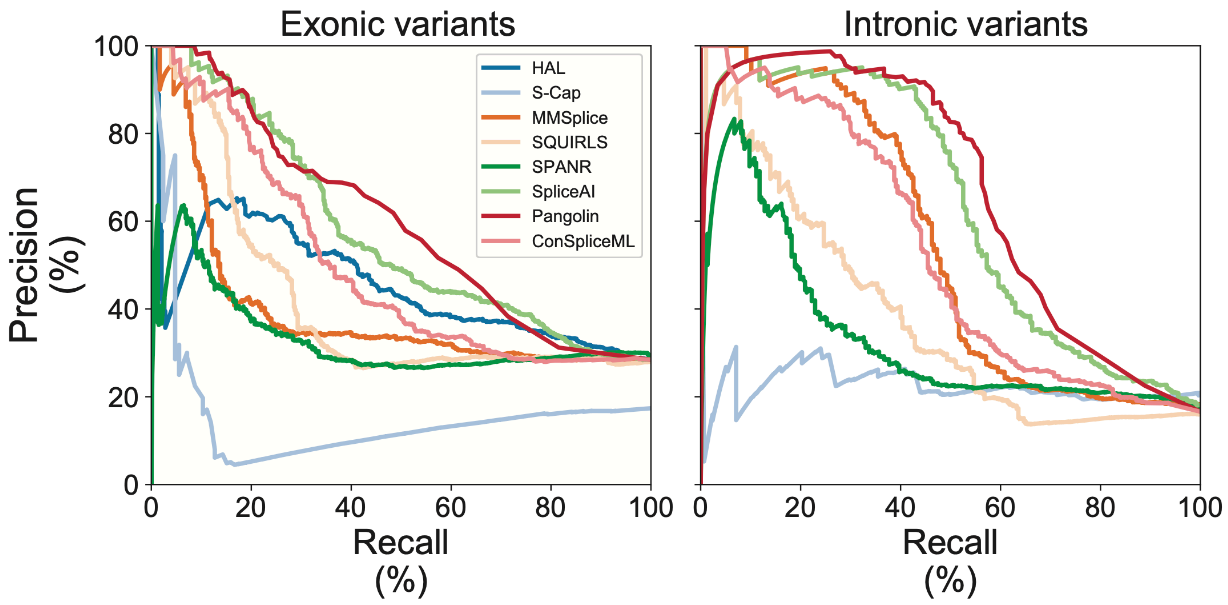

Supplementary Figure 10. Classification performance without essential splice site mutations. Precision-recall curves showing algorithms' performance at distinguishing SDVs from splicing-neutral variants in each dataset, for exonic variants (identical to **Figure 3C**) and intronic variants after removing variants at essential splice sites.

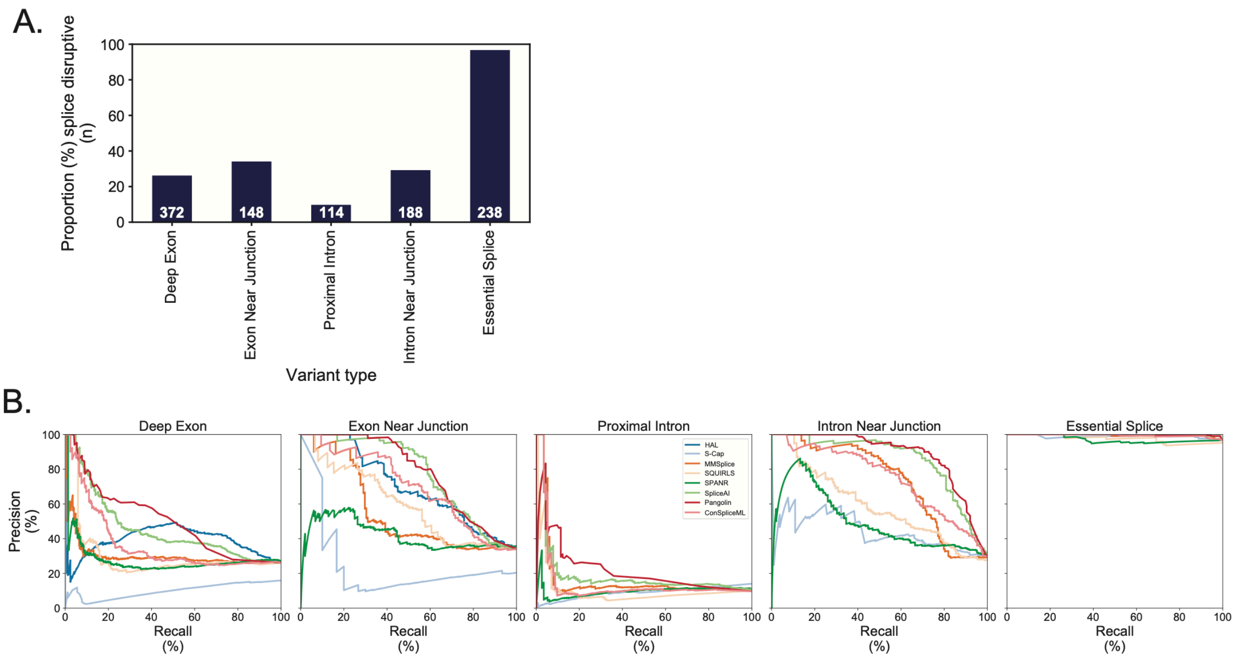

Supplementary Figure 11. Classification performance by variant category. (A) Proportion of variants which are splice disruptive by variant category. Counts of splice disruptive variants in each category are inset. **(B).** Precision-recall curves showing algorithms' performance at distinguishing SDVs from splicing-neutral variants in each variant category as defined in **Figure 1B**.

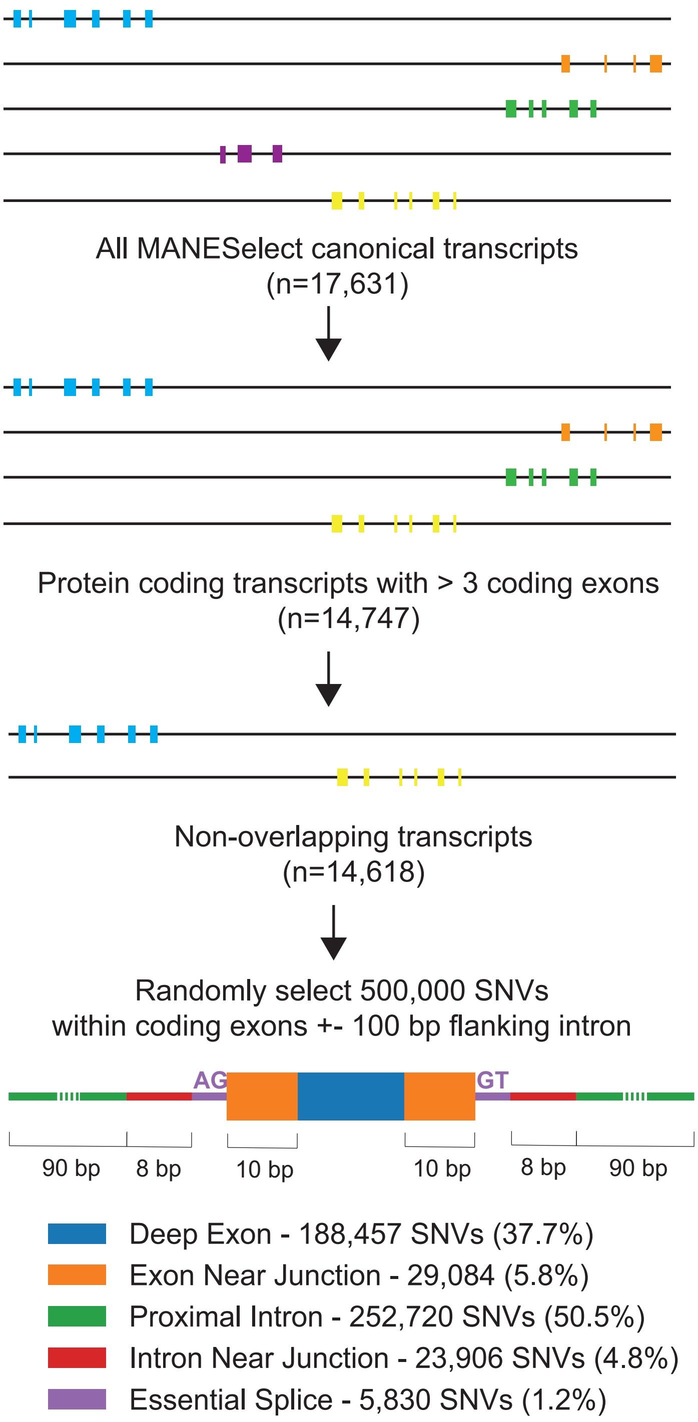

Supplementary Figure 12. Background set of random exonic and near-exonic variants. Schematic shows criteria used to select gene models and counts of MANESelect transcripts remaining at each step. At bottom, counts and proportions of background set variants by category.

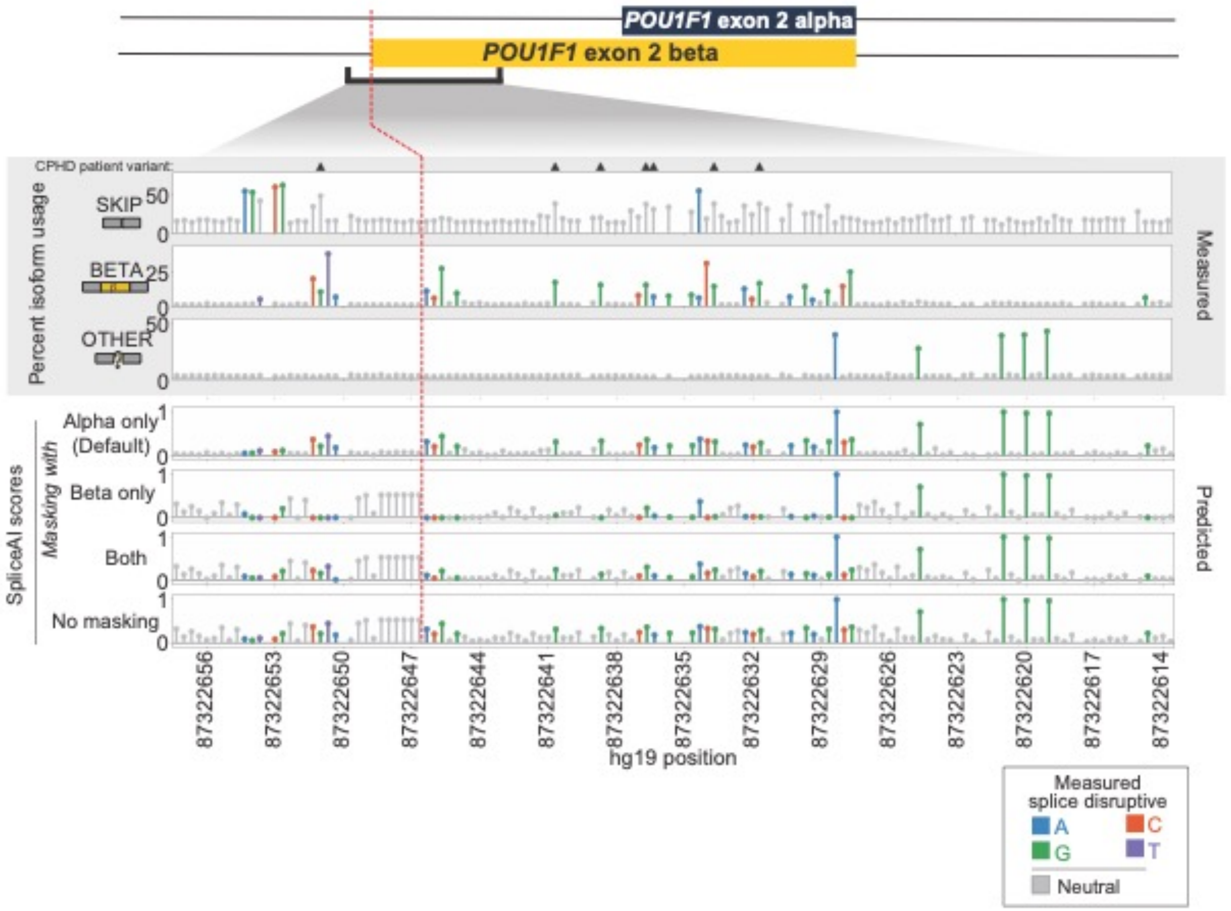

Supplementary Figure 13. Effects of gene model annotation on SpliceAI predictions near *POU1F1* exon 2 beta acceptor. MPSA measured percent usage of *POU1F1* isoforms are shown in the upper tracks (gray background), with variants called SDVs shaded with color and denoted as in **Supplementary Figure 4**. SpliceAI deltaMax scores are shown in the bottom three tracks, masked obtained using default annotation (alpha isoform only; top), beta isoform only (second), both isoforms (third), or without masking (bottom). Combined pituitary hormone deficiency (CPHD) patient variants are marked with black triangles.

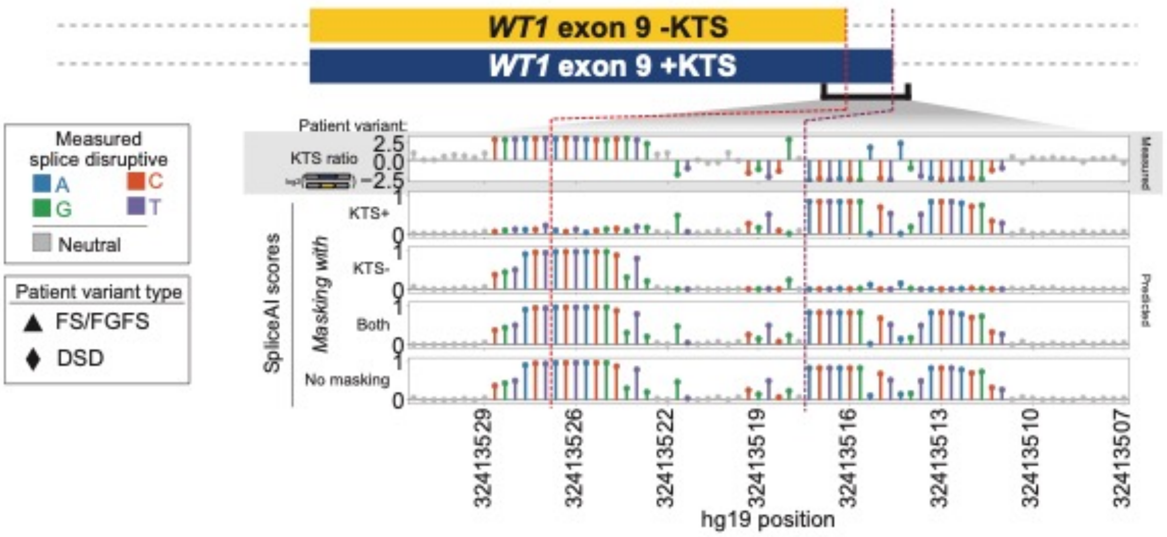

Supplementary Figure 14. Effects of gene model annotation on SpliceAI predictions at *WT1* exon 9 alternate donors. MPSA measured log_2ratio of *WT1* exon 9 alternate isoforms (top panel) along with masked SpliceAI predictions scored with annotation files of the KTS+ isoform (second track), KTS- isoform (third), both isoforms (fourth), and unmasked scores (bottom track) by variant position (x-axis). Gray lollipops denote MPSA measured splicing-neutral variants, while shaded lollipops indicate the base pair change of each measured SDV (dark colors). Pathogenic Frasier's syndrome (FS) and focal segmentation glomerulosclerosis (FSGS) clinical variants are shown with black triangles, and variants observed in individuals with 46,XX ovotesticular differences in sexual development (OTDSD) are denoted by black diamonds.

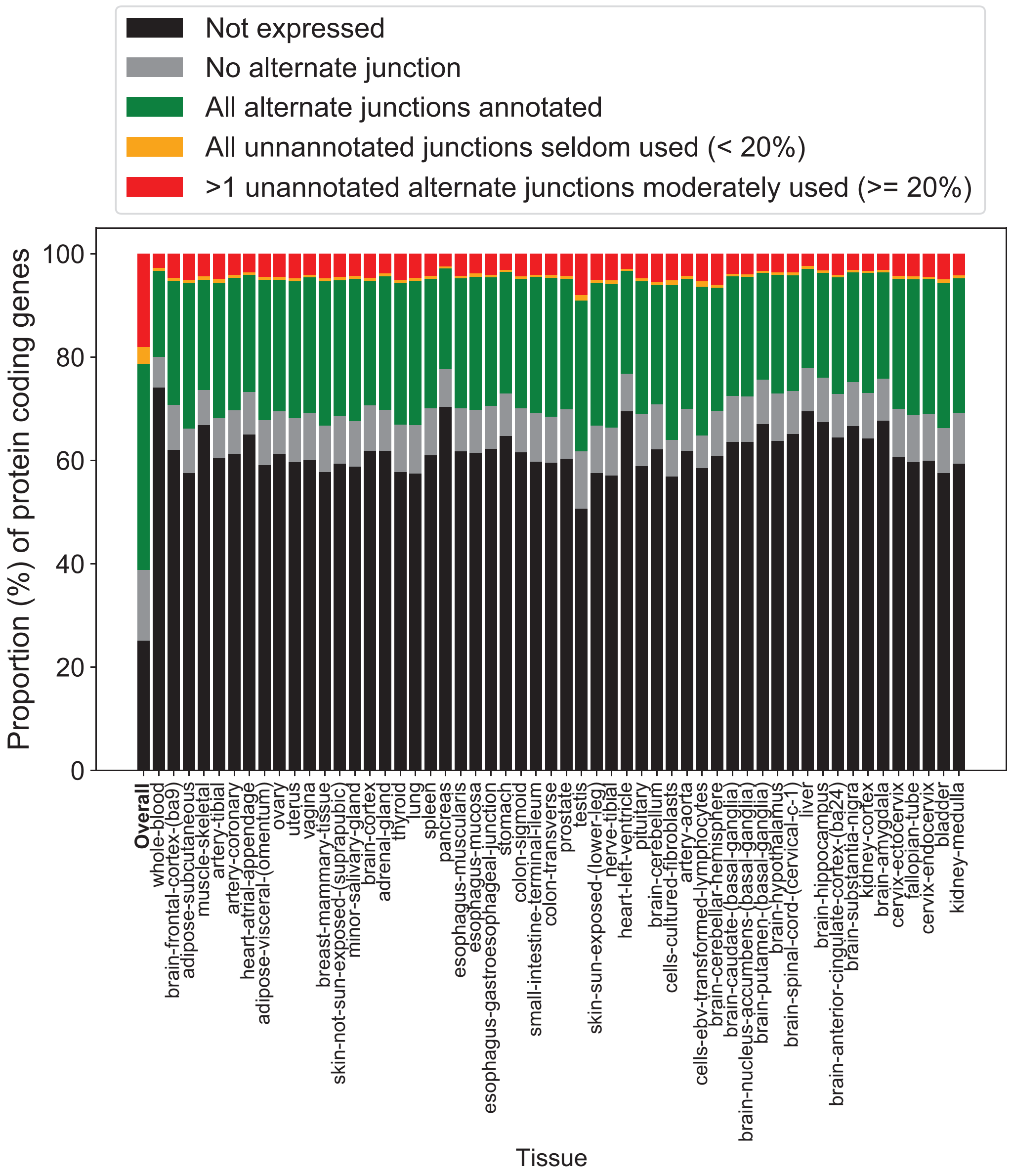

Supplementary Figure 15. Annotation sensitive alternatively spliced genes. Proportion of protein coding genes within GTEx (y-axis) that are either not expressed (CPM < 0.1; black), have no expressed alternate splice junctions (gray), have all alternatively used splice junctions present in SpliceAI annotations (green), have only seldom used unannotated alternate splice junctions (orange), or have at least one unannotated alternate splice junction with at least modest use (≥ 20%; red). Proportions are shown across all tissues (first bar labeled 'overall') and within individual tissues.
