# Supplementary material for: Benchmarking splice variant prediction algorithms using massively parallel splicing assays
Source: Genome Biol. 2023 Dec 21;24:294. doi: 10.1186/s13059-023-03144-z (PMC10734170; doi:10.1186/s13059-023-03144-z)
Supplement: Supplementary file 5 — Additional file 5. Review history. [file 13059_2023_3144_MOESM5_ESM.docx]

Review History

**First round of review**

**Reviewer 1**

**Were you able to assess all statistics in the manuscript, including the appropriateness of statistical tests used?**

Yes, and I have assessed the statistics in my report.

**Comments to author:**

In this work, Smith and Kitzman assess the performance of 8 algorithms for predicting the effect of mutations on splicing outcome using 6 datasets: 5 involving MPSA + 1 they curated. The work supplements recent publications that focused on assessing such algorithms on other large scale assays and/or curated datasets that focused on clinically relevant variants. As the authors point out, those other datasets/publications focus on clinically relevant variants and thus (a) are heavily biased towards what researchers were already able to detect (e.g. splice site disruption) and (b) assays with only few variants per exon but hundreds of exons. Here, the authors only assess 6 exons, but hundreds of variants for each. The authors break down performance across regions and types (intronic/exonic), test whether the algorithms agree between themselves (e.g. SpliceAI and Pangolin, which is based on SpliceAI, are almost identical), and highlight areas for future improvement (deep intronic/exonic, context specific calibration, etc.)

Overall there is much to like in this work. The writing is clear, the analysis is elaborate, the figures are mostly well done. Maybe most refreshing is the fact this does not read as yet another CS/ML bioinformatics work - while the authors perform the expected performance analysis, they are also careful to look into the effect of genome annotation, highlight cases of interest, then assess the relevance of that across GTEX. With that said, there are several issues that need to be addressed.

We will start with the premise itself. Conceptually, we argue there are two/three related but distinct tasks that the authors mix here: One is to compute some metric of splicing outcome. The related line of works can be traced back to the first "splicing code" model (Barash et al 2010) where a machine learning model predicts outcome such as "splice site usage" as in Pangolin (which is trained using SpliSER's output), or PSI as in MMSplice and SPANR (the latter predicts only PSI low/med/high but across tissues). The second is to predict deleterious effects. Algorithms for this task may use additional features such as the affected protein domain, which are irrelevant for the former. There are specific tools for detecting such effects which should be at the very least mentioned here, such as: regSNPs (Zhang et al 2017) which focuses on synonyms mutations (exactly those the authors highlight here as requiring more attention), IntSplice (Shibata et al 2016), SPiCE (Leman et al 2018), TraP (Gelfman et al 2018). Note that all of the above pre-dates the deluge of DL based models and focus squarely on deleterious variants (with a focus on splicing). Finally, there is the task of detecting splice sites, including mutations that create/disrupt those. That third task is the original focus/target function of SpliceAI. However, these three tasks are clearly related and much of the applications of the aforementioned tools is for detecting deleterious mutations even when they were not originally developed/trained for that. The authors would do well to reframe the tasks and the tools accordingly.

Furthermore, the above distinction between tasks have implications for the analysis of tools performed here: The analysis focuses on the effect of many mutations around a few exons. Therefore it is inherently limited: Most of the mutations may not be clinically relevant while the analysis involves only a few exons. The authors are right to point out the deficiencies of focusing solely on clinically relevant mutations, as these are likely highly biased. Nonetheless, we don't really know what is the search space/characteristics of clinically relevant mutations. Thus it makes sense to also assess the tools on datasets such as those included here. Again, the authors would do well to highlight this as a point of discussion with a more balanced/transparent view of the limitations of each approach/data.

A related point to the above, which affects the analysis performed here, is that tools like SpliceAI and Pangolin use the canonical splice sites mask to highlight deleterious mutations. But the MPSA are not about deleterious mutations but rather the effect of mutations on splicing outcome in general. Thus, one could argue that a more fair comparison to other tools in 5/6 datasets based on MPSA is to remove the annotation and assess the predictions of these tools without them. At the very least, include their performance w/wo annotation. In general, it would have been good to see more analysis of the effect of annotation on tools like SpliceAi/Pangolin in transcriptome wide analysis, as this seems to be an overlooked issue which deserves to be addressed (e.g. in one of the main figures).

Another limitation of this study/analysis which should be brought up in the discussion is the lack of condition specificity. Most models they test are not condition/tissue specific, while some (e.g. SPANR, PANGOLIN) were not trained on the conditions in which the experiments were done (and, as the authors do note nicely page 7 line 30, there may be differences between the native cell line and the reporter assay).

It was not clear how neutral vs SDV is defined. The authors note it was done according to the classification made by each study (page 8 line 9), but clearly that can affect the results here and may vary between studies. At least a discussion of that point in the main text is warranted.

Another point to clearly state in the discussion is the limitation in assessing deep intronic regions (one of the highlights of SpliceAI) and tissue specificity effects (where PANGOLIN claims to improve upon SpliceAI).

A highly relevant work recently published is that by Wagner et al (Nat Gen 2023) which the authors should address.

General question: Did the authors look at whether the *direction* of change is correct (increase/decrease) or just the magnitude? That should be included in the analysis/explained in the main text as well.

The authors should include not only the final results in the format of excel sheets etc. but also a code that can be used to reproduce the results/figures and detailed report of how each tool was run (parameter setting). This comment is actually more general than reproducibility and relates to the impact/significance of this work: As is, the paper is limited to the specific data/tools analyzed and the specific results of that analysis. But if the authors were to release this work as a package where researchers can install the tools and then run them on data in a given format to generate the kind of plots/figures included here (e.g. Fig2a is a nice/useful visualization, or prAUCs as in Fig3) that would be much more useful - researchers could run these tools on additional datasets and/or add tools to easily compare against the tools included here.


Minor comments:
Technically one would argue SMN2 exon 7 exclusion is not a "defect" since it's the normal/WT (p. 2 line 20).

Page 10 line 16 "sklearn" → scikit-learn (and please cite it, as explained here: https://scikit-learn.org/stable/about.html)

Fig2: Eyeballing the data in Fig2a looks like for ALPAExon region SPANR is doing very well yet not in Fig2b summary. Regardless, having visual representation of hit/miss in Fig2a and numbers in 2b would be helpful. Are colors/nucleotides per position given only for the strongest effect? Is agreement between experiments and predictions per position computed by the max effect per position regardless of the actual change of bases?

Fig4C:
It seems that HAL is doing significantly better on exonic variants on several datasets (BRCA1, MLH1, POU1F1, even RON) for the high confidence predictions. This seems worth noting. In several cases HAL's ROC curve completely flattens, which is suspicious. Maybe that is because there are variants in the plot for which the algorithm does not report any output? If that's the case the plots should be adjusted - it's not "fair" to plot a bad ROC over variants the algorithm doesn't report. Instead each ROC should have only the set it reports and then the number (n) of variants tested for each algorithm either as an inset bar graph or in the legend.

Fig5:
These results are unclear. How can the non optimized threshold, recommended in the original publication, give better performance than the optimized threshold?? Clearly there are points in this graph that are BELOW that dashed line… Is this dashed line over all datasets combined? averaged? weighted average? Why not show the effect of optimization per dataset?

**Reviewer 2**

**Were you able to assess all statistics in the manuscript, including the appropriateness of statistical tests used?**

No, I do not feel adequately qualified to assess the statistics.

**Comments to author:**

This analysis shows that two methods based on annotated splice sites, SpliceAI and Pangolin, are better at predicting results from massively parallel splicing assays.

I have a few minor points. Firstly, it is not made clear what annotated splice sites refers to when discussing the masking of splice AI and Pangolin. It seems to me in the default cases, annotated splice sites are those in the MANE Select transcript. Alternative transcript splice sites are only added when investigating the effect on alternative isoforms of POU1F1 and WT1. If this is true, this needs to be made more clear. Generally "annotated" splice sites would be assumed to refer to all splice sites in a gene set, which in the case of Ensembl WT1 would extend to 8 coding transcript, 2 NMD transcript and 12 non-coding variants.

All genes in the study are named with their HGNC names except RON, which is officially called MST1R. If the authors continue to use RON, they should at least reference MST1R when first introduced.

The authors should also take care over the use of "isoform". Most of the time it is acceptable, but occasionally they confuse it with the idea of "alternative transcript", eg "against two alternative isoforms (ENST00000358487.10 for FGFR2c and ENST00000358487.5 for FGFR2b", where "transcript" needs to be used, not isoform.

The phrase "a small amount of missingness" doesn't sound particularly scientific ....

MANESelect is actually "MANE Select"

The problem of isoform selection as highlighted by the authors: "Our results also highlight the major influence of gene model annotation, a required input for many splice effect predictors ... Moreover, they may not be possible when the functionally relevant isoforms are not known in advance." is important. Obviously, for individual genes nothing is going to work better than research, but beyond that as far as I know there is only one method that can distinguish functionally relevant isoforms, and that is TRIFID. TRIFID is used to score alternative splice isoforms with annotated pathogenic variants here: <https://pubmed.ncbi.nlm.nih.gov/36257961/>

It is probably not relevant for this paper because there are so few genes, but it would be interesting to whether it would be useful to include splice sites from transcripts with high scoring TRIFID scores instead of just the MANE Select transcript.

The authors use transcript expression as a proxy for functional relevance when calculating the number of important alternative transcripts. It is worth point¡ng out that conservation is probably just as important if not more important. Of the three splice events mentioned in the paper, the distinct acceptors in POU1F1 are conserved in mammals, the distinct donors in WT1 are annotated in fish, and the the mutually exclusively spliced exons in FGFR2 have a last common ancestor in chordates.

**Authors Response**

**Point-by-point responses to the reviewers’ comments:**

Reviewer #1: In this work, Smith and Kitzman assess the performance of 8 algorithms for predicting the effect of mutations on splicing outcome using 6 datasets: 5 involving MPSA + 1 they curated. The work supplements recent publications that focused on assessing such algorithms on other large scale assays and/or curated datasets that focused on clinically relevant variants. As the authors point out, those other datasets/publications focus on clinically relevant variants and thus (a) are heavily biased towards what researchers were already able to detect (e.g. splice site disruption) and (b) assays with only few variants per exon but hundreds of exons. Here, the authors only assess 6 exons, but hundreds of variants for each. The authors break down performance across regions and types (intronic/exonic), test whether the algorithms agree between themselves (e.g. SpliceAI and Pangolin, which is based on SpliceAI, are almost identical), and highlight areas for future improvement (deep intronic/exonic, context specific calibration, etc.)

Overall there is much to like in this work. The writing is clear, the analysis is elaborate, the figures are mostly well done. Maybe most refreshing is the fact this does not read as yet another CS/ML bioinformatics work - while the authors perform the expected performance analysis, they are also careful to look into the effect of genome annotation, highlight cases of interest, then assess the relevance of that across GTEX. With that said, there are several issues that need to be addressed.

We will start with the premise itself. Conceptually, we argue there are two/three related but distinct tasks that the authors mix here: One is to compute some metric of splicing outcome. The related line of works can be traced back to the first "splicing code" model (Barash et al 2010) where a machine learning model predicts outcome such as "splice site usage" as in Pangolin (which is trained using SpliSER's output), or PSI as in MMSplice and SPANR (the latter predicts only PSI low/med/high but across tissues). The second is to predict deleterious effects. Algorithms for this task may use additional features such as the affected protein domain, which are irrelevant for the former. There are specific tools for detecting such effects which should be at the very least mentioned here, such as: regSNPs (Zhang et al 2017) which focuses on synonyms mutations (exactly those the authors highlight here as requiring more attention), IntSplice (Shibata et al 2016), SPiCE (Leman et al 2018), TraP (Gelfman et al 2018). Note that all of the above pre-dates the deluge of DL based models and focus squarely on deleterious variants (with a focus on splicing). Finally, there is the task of detecting splice sites, including mutations that create/disrupt those. That third task is the original focus/target function of SpliceAI. However, these three tasks are clearly related and much of the applications of the aforementioned tools is for detecting deleterious mutations even when they were not originally developed/trained for that. The authors would do well to reframe the tasks and the tools accordingly.

We thank the reviewer for the clarity in distinguishing these tasks. We agree that each scorer (and the MPSA data) maps to a distinct one of these. We have updated the manuscript in several places to clarify this:
- added a new paragraph to the discussion, and revised another, to emphasize the differences between these tasks, and the fact that each tool does only one of them, in particular, that most of the tools model a quantitative and/or qualitative splicing or splice site usage change, which is not the same as predicting a pathogenic effect (lines 332-337)
- added material to the background/introduction setting forth the idea of the splicing code (line 60)
- added citations to the studies and tools the reviewer mentions, which we agree are relevant to these distinctions.

Furthermore, the above distinction between tasks have implications for the analysis of tools performed here: The analysis focuses on the effect of many mutations around a few exons. Therefore it is inherently limited: Most of the mutations may not be clinically relevant while the analysis involves only a few exons. The authors are right to point out the deficiencies of focusing solely on clinically relevant mutations, as these are likely highly biased. Nonetheless, we don't really know what is the search space/characteristics of clinically relevant mutations. Thus it makes sense to also assess the tools on datasets such as those included here. Again, the authors would do well to highlight this as a point of discussion with a more balanced/transparent view of the limitations of each approach/data.

We agree that with additional saturation MPSAs will come the opportunity to move beyond the present limitations from having only a few exons’ worth of saturation data. We have revised the limitations paragraph to prominently mention this and suggest ways to mitigate the impact in the future (line 375, “Our conclusions are limited by …”)

A related point to the above, which affects the analysis performed here, is that tools like SpliceAI and Pangolin use the canonical splice sites mask to highlight deleterious mutations. But the MPSA are not about deleterious mutations but rather the effect of mutations on splicing outcome in general. Thus, one could argue that a more fair comparison to other tools in 5/6 datasets based on MPSA is to remove the annotation and assess the predictions of these tools without them. At the very least, include their performance w/wo annotation. In general, it would have been good to see more analysis of the effect of annotation on tools like SpliceAi/Pangolin in transcriptome wide analysis, as this seems to be an overlooked issue which deserves to be addressed (e.g. in one of the main figures).

We agree that annotation can have an outsized impact upon splice effect predictor results. We have additionally documented these impacts by:
(A) using our 500k variant background set to quantify, genome-wide, the degree to which predictions change with vs without masking for SpliceAI and Pangolin, or using different annotations (presented only for Pangolin due to higher GPU compute time required for SpliceAI, which as we showed elsewhere is likely very similar based upon these algorithms’ overall very highly correlation). This is presented in a new main Figure 6 which includes the FGFR2 example (previously Supplementary Figure 16).
(B) Updated Supplementary Figures 13 and 14 to show variant scores with masking disabled, in addition to the existing tracks in which the annotations used for masking were updated.

We note that some of the other tools implicitly apply masking relative to the targeted exon, in the sense that loss of other (unannotated) splice sites does not score highly, and variants that strengthen the targeted sites also score as neutral (i.e., indistinguishable from non-effect variants). A key difference is that, unlike SpliceAI/Panoglin, in other tools, this behavior is not customizable and in some cases, the annotations could not readily be changed (e.g., SQUIRLS does not offer this as a command line option). For this reason, we have restricted our examination of annotation effects to primarily SpliceAI/Pangolin.

Another limitation of this study/analysis which should be brought up in the discussion is the lack of condition specificity. Most models they test are not condition/tissue specific, while some (e.g. SPANR, PANGOLIN) were not trained on the conditions in which the experiments were done (and, as the authors do note nicely page 7 line 30, there may be differences between the native cell line and the reporter assay).

We have noted this limitation and referenced a few newer tissue-specific splice effect predictors (line 385, “In the future, using more physiologically relevant cell lines…”)

It was not clear how neutral vs SDV is defined. The authors note it was done according to the classification made by each study (page 8 line 9), but clearly that can affect the results here and may vary between studies. At least a discussion of that point in the main text is warranted.

We thank the reviewer for raising this point. Indeed, we attempted to find a consistent threshold (i.e., pvalue, log2fc) to define SDVs across different groups’ MPSA/SGE studies of different genes. In the end we were not able to develop such a threshold to our satisfaction. We believe this reflects that the MPSA approaches are not nearly as standardized as assays such as RNA-seq (while noting even this commodity assay still suffers from significant batch effects within and between labs). In this case, differences in data generation, processing, and reporting were complicating factors (e.g., some MPSA studies do not report p-values/z scores). Moreover, it was not even clear that a single consistent threshold should exist: the threshold of a splicing change that is functionally impactful may differ between different exons or genes, or even among different isoforms of a single gene. In absence of a consistent thresholding strategy, we decided to follow the intuition of the respective MPSA studies’ authors and take their disruptive/neutral calls as originally reported. We have added a note to emphasize this to the main text (line 126, “Splice-disruptive variants (SDVs), and conversely,…”)

Another point to clearly state in the discussion is the limitation in assessing deep intronic regions (one of the highlights of SpliceAI) and tissue specificity effects (where PANGOLIN claims to improve upon SpliceAI).

We agree that deep intronic regions are an important source of SDVs. To our knowledge there is not yet a large-scale experimental screen of variants in these regions, probably owing to their cumulative size and the resulting low density of SDVs. We have noted this in the discussion (line 379…, “We did not include deep intronic variants…”) Tissue specific splicing effects are another key factor in splice effect predictions, and understanding which algorithm best performs at this task is an important question to address. However, since mini-gene assays typically use cancer immortalized cell lines, we do not think that MPSA data would be the best method to benchmark the tissue specificity of the given tools. So this is a limitation of our study. We have highlighted this point in the discussion (line 383, “However, as with MPSAs, those typically rely..”).


A highly relevant work recently published is that by Wagner et al (Nat Gen 2023) which the authors should address.

We agree and have added several lines to the discussion citing this very recent paper (line 368, “Recently, a new tool called SpliceMap…”)

General question: Did the authors look at whether the *direction* of change is correct (increase/decrease) or just the magnitude? That should be included in the analysis/explained in the main text as well.

We agree that it is important to look at direction of change, and we do report correlation statistics, which capture this, in Supplementary Figure 9. To complement these, the remaining analyses used algorithms scores’ absolute values and similarly transformed MPSA data. We felt reporting correlation statistics alone could obscure some important features for the following reasons:

(1) Several of the MPSA datasets (WT1, RON, POU1F1) provided, for each variant, a vector of usage fractions for all possible junctions/isoforms. Each isoform has a different baseline mean and variance, so that taking a correlation against any one might not be informative. Moreover, since isoform/junction fractions sum to 1, any given variant perturbed isoforms in opposite directions, making it difficult to ‘pick’ an isoform for which to represent direction of effect. Therefore, we opted to take as each variant’s score the highest-magnitude isoform specific z-scaled score (or difference in usage fraction, when z scores weren’t available), across isoforms.
(2) As the reviewer noted earlier, algorithms differed by the classification task and scores’ meanings. Scores were not directly analogous to what the MPSA provides, namely a measure of the (differential) usage across the many possible isoforms. Because of the scores’ different scales and interpretations, we felt the quantitative common denominator was the (predicted) likelihood of a change in either direction vs the magnitude of change as measured – provided for the most uniform comparison among them algorithms.
(3) Finally, we appreciate how considering not only the magnitude but also direction could be especially important for exons that have a moderate level of inclusion at baseline, as these shift more readily towards either greater or lesser inclusion, an effect of the ‘scaling law’ of splicing (Baeza-Centurion et al, 2019). Among the MPSA data we used, the only exon with moderate baseline inclusion was FAS. Therefore, specifically for FAS, we calculated correlations between the that MPSA’s measured signed delta-PSI scores and the signed scores (for algorithms that reported a signed score); those correlations are in Supplementary Figure 9. For the other exons, most changes were in the direction of less wildtype inclusion so that there were few increases of exon inclusion, and similarly, algorithms would mask out predictions in the direction of increased inclusion.

The authors should include not only the final results in the format of excel sheets etc. but also a code that can be used to reproduce the results/figures and detailed report of how each tool was run (parameter setting). This comment is actually more general than reproducibility and relates to the impact/significance of this work: As is, the paper is limited to the specific data/tools analyzed and the specific results of that analysis. But if the authors were to release this work as a package where researchers can install the tools and then run them on data in a given format to generate the kind of plots/figures included here (e.g. Fig2a is a nice/useful visualization, or prAUCs as in Fig3) that would be much more useful - researchers could run these tools on additional datasets and/or add tools to easily compare against the tools included here.

We have posted a github repository with jupyter/ipython notebooks needed to reproduce figures and analyses from the manuscript: https://github.com/kitzmanlab/splicebench2023/ and https://github.com/kitzmanlab/splfxseq . A snapshot of this code as well as the data files needed are posted on Zenodo at: https://zenodo.org/record/8351879 . We have noted several places within the primary notebook (main_mss_plots.ipynb) where a user could make modifications in order to either (a) evaluate additional algorithms vs the same benchmark variants or (b) incorporate additional benchmark sets, e.g., from future MPSA. We hope this will be useful to the community.

Minor comments:
Technically one would argue SMN2 exon 7 exclusion is not a "defect" since it's the normal/WT (p. 2 line 20).

We have rephrased this passage, removing term “defect” (line 55)

Page 10 line 16 "sklearn" → scikit-learn (and please cite it, as explained here: https://scikit-learn.org/stable/about.html)

Added

Fig2: Eyeballing the data in Fig2a looks like for ALPAExon region SPANR is doing very well yet not in Fig2b summary. Regardless, having visual representation of hit/miss in Fig2a and numbers in 2b would be helpful. Are colors/nucleotides per position given only for the strongest effect? Is agreement between experiments and predictions per position computed by the max effect per position regardless of the actual change of bases?

We have attempted to clarify Figure 2b by revising the legend (line 594 “… concordance between each algorithm’s binary classification…”). We picked a single threshold since to reflect what would be done in practice, when the boundaries between regions with different densities of ground-truth splicing constraint are not known up front. Also, we felt that taking per-region correlation/concordance might unfairly benefit algorithms with poor global concordance as illustrated below:


Fig4C:
It seems that HAL is doing significantly better on exonic variants on several datasets (BRCA1, MLH1, POU1F1, even RON) for the high confidence predictions. This seems worth noting. In several cases HAL's ROC curve completely flattens, which is suspicious. Maybe that is because there are variants in the plot for which the algorithm does not report any output? If that's the case the plots should be adjusted - it's not "fair" to plot a bad ROC over variants the algorithm doesn't report. Instead each ROC should have only the set it reports and then the number (n) of variants tested for each algorithm either as an inset bar graph or in the legend.

We thank the reviewer for pointing this out. We investigated the odd shape of the HAL curves noted in Fig4C. We confirmed that this did not arise due to inclusion of unscored datapoints. Within the background set of variants (~500k, of which ~220k are exonic and therefore scored by HAL), we observed that HAL’s scores (specifically, |mut PSI – wt PSI| ) had a nearly unform distribution. This struck us as biologically implausible. In addition, these scores were confined to the range [0,50], dictated by our use of an uninformative 50% WT PSI (a parameter which HAL requires). The differences in ranges of the background set variants and MPSA variants’ scores lead them to be not directly comparable so we have removed HAL from this analysis (Figure 4B and 4C). This issue was specific to only one algorithm (HAL) within the ‘transcriptome-adjusted sensitivity’ analysis; HAL’s prAUC analyses were unaffected, and no other algorithm was similarly affected in this analysis.


Fig5:
These results are unclear. How can the non optimized threshold, recommended in the original publication, give better performance than the optimized threshold?? Clearly there are points in this graph that are BELOW that dashed line… Is this dashed line over all datasets combined? averaged? weighted average? Why not show the effect of optimization per dataset?

We agree this was not clearly expressed. By ‘optimal’, we meant the per-algorithm score that maximized Youden’s J for a given dataset, or category of variants. This may or may not be globally better (by some metric) than the authors’ original cutoff. The point of this figure was to illustrate that there is no single individual cutoff which is globally best by a defined metric (Youden’s J in this case) across all datasets. We have revised the figure legend to better explain this (line 589)


Reviewer #2: ===

This analysis shows that two methods based on annotated splice sites, SpliceAI and Pangolin, are better at predicting results from massively parallel splicing assays.

I have a few minor points. Firstly, it is not made clear what annotated splice sites refers to when discussing the masking of splice AI and Pangolin. It seems to me in the default cases, annotated splice sites are those in the MANE Select transcript. Alternative transcript splice sites are only added when investigating the effect on alternative isoforms of POU1F1 and WT1. If this is true, this needs to be made more clear. Generally "annotated" splice sites would be assumed to refer to all splice sites in a gene set, which in the case of Ensembl WT1 would extend to 8 coding transcript, 2 NMD transcript and 12 non-coding variants.

Indeed, gene annotations used by default differed widely among the included tools: Pangolin and MMSplice do not come with any annotation, and require the user to supply a gtf file (which in the case of Pangolin is then converted to a custom file format). SpliceAI provides gencode v24 canonical transcripts as a default and can accept a custom annotation in UCSC refFlat-like format. SQUIRLS ships with pre-built UCSC, RefSeq, ENSEMBL annotations (though the version is unclear and there is no straightforward option to input custom annotations).

For the benchmark set variant genes (WT1, MST1R, MLH1, FAS, BRCA1), we used only the MANE Select annotation, and held this constant across tools, to enable fair comparison of each tool without variability due to differences in annotation used. We have revised the text to emphasize this choice (line ~470, “To ensure that differences between tools predictions’…”)

All genes in the study are named with their HGNC names except RON, which is officially called MST1R. If the authors continue to use RON, they should at least reference MST1R when first introduced.

We thank the reviewer for noting this discrepancy. We have updated the manuscript and main figures to use MST1R instead of RON (the name used in the 2018 publication from which we obtained the splicing assay data)

The authors should also take care over the use of "isoform". Most of the time it is acceptable, but occasionally they confuse it with the idea of "alternative transcript", eg "against two alternative isoforms (ENST00000358487.10 for FGFR2c and ENST00000358487.5 for FGFR2b", where "transcript" needs to be used, not isoform.

We have updated the manuscript accordingly

The phrase "a small amount of missingness" doesn't sound particularly scientific ....

We have rephrased this

MANESelect is actually "MANE Select"

We have updated the manuscript accordingly

The problem of isoform selection as highlighted by the authors: "Our results also highlight the major influence of gene model annotation, a required input for many splice effect predictors ... Moreover, they may not be possible when the functionally relevant isoforms are not known in advance." is important. Obviously, for individual genes nothing is going to work better than research, but beyond that as far as I know there is only one method that can distinguish functionally relevant isoforms, and that is TRIFID. TRIFID is used to score alternative splice isoforms with annotated pathogenic variants here: https://pubmed.ncbi.nlm.nih.gov/36257961/
It is probably not relevant for this paper because there are so few genes, but it would be interesting to whether it would be useful to include splice sites from transcripts with high scoring TRIFID scores instead of just the MANE Select transcript.

We were not aware of this tool, and thank the reviewer for pointing it out. Indeed, TRIFID ranks highly several of the relevant alternative isoforms discussed in our study, though the problem of whether to include or exclude them from the masking remains unclear in the general case. We have mentioned TRIFID and added a citation in the relevant section (lines ~363….)

The authors use transcript expression as a proxy for functional relevance when calculating the number of important alternative transcripts. It is worth point¡ng out that conservation is probably just as important if not more important. Of the three splice events mentioned in the paper, the distinct acceptors in POU1F1 are conserved in mammals, the distinct donors in WT1 are annotated in fish, and the the mutually exclusively spliced exons in FGFR2 have a last common ancestor in chordates.

We agree, and note that conservation is one of the primary features integrated into TRIFID’s model, which seems to be an ideal way to identify functionally relevant alternative transcripts.

**Second round of review**

**Reviewer 1**

We are happy with the authors response, edits, and additions. We specifically liked the new Fig6 addition. We congratulate them on a work well done and are sure their work will be highly useful for the community.

We have only one standing issue we think would be easy for the authors to address before publication:
As the authors note, the various studies they collected used different experimental methods/settings. In the manuscript the authors state they have used the original authors “thresholds” to decide on change vs no change. Clearly, this is a spectrum. While their answer indicates they have wrangled the data into the above classifications, it is not immediately clear how that was done. The authors should include the needed information i.e. the original labels and how these were translated into labels in supp methods. Presumably, it would be a vector of inclusions/usage by some statistic, the matching one for the WT, and then how the statistics were defined to make a change/no change call. We assume all this data/pipeline is already available, the authors just need to include it in their repositories/tables which should be straightforward. This can help not only in reproducing their results but also testing the effect of the thresholds they used here (we understand such testing will take long if the authors were to start testing different combinations and we don’t see such additional testing over the defaults as mandatory for getting this work published - just include the info for reproducibility and clarity).

Other two minor technical comments:
The version we downloaded from the journal website was somewhat scrambled with line numbering overlapping (one column for all lines, then another one overlapping the text per page) and the edits clearly shown. No “clean” version was included. This made reading the revision harder. We also noticed that some of the figures were in good quality while others were in poor quality. Again, we suspect this is the result of some conversion on the journal website, but it would be important to make sure all figures look good in a “clean” version. With that in mind, we only found one error: “Together, there our results” in what seems to be line 330 assuming the line numbers are correct.

**Reviewer 2**

All suggestions have been answered

**Authors Response**

**Point-by-point responses to the reviewers’ comments:**

We thank the reviewers for their thorough reading and feedback on our revised manuscript. We have made the requested formatting changes, and adopted the editor’s suggested changes to our abstract.

Reviewer 1 requested additional detail on how we transformed scores from different MPSA studies into classifications of neutral/splice disruptive/intermediate. We have significantly expanded the description of that preprocessing under the heading ‘Saturation mutagenesis datasets’ within the Methods section. By and large we simply took the existing classifications from those previous studies’ supplementary materials as-is. In a few cases we filtered to specific subsets of variants, and the justification for these choices is described in that paragraph. For one study (MST1R/RON/Braun et al), classifications of splice disruptive vs neutral as described in that manuscript were not provided in their supplementary information, so we reimplemented their significance testing methods as described and arrived at the same number of SDVs as they did. We have added to our github repo a ipython notebook with the code implementing these processing steps (<https://github.com/kitzmanlab/splicebench2023/blob/main/notebooks/ron_snvs_signif_testing.ipynb>)
